# Targeting ERK-Hippo Interplay in Cancer Therapy

**DOI:** 10.3390/ijms21093236

**Published:** 2020-05-03

**Authors:** Karel Vališ, Petr Novák

**Affiliations:** Laboratory of Structural Biology and Cell Signaling, Institute of Microbiology of the Czech Academy of Sciences, 14220 Prague, Czech Republic

**Keywords:** MAPK, ERK, Hippo, MST, PI3K, YAP, cancer, apoptosis, caspase, inhibitors, natural compounds, therapy

## Abstract

Extracellular signal-regulated kinase (ERK) is a part of the mitogen-activated protein kinase (MAPK) signaling pathway which allows the transduction of various cellular signals to final effectors and regulation of elementary cellular processes. Deregulation of the MAPK signaling occurs under many pathological conditions including neurodegenerative disorders, metabolic syndromes and cancers. Targeted inhibition of individual kinases of the MAPK signaling pathway using synthetic compounds represents a promising way to effective anti-cancer therapy. Cross-talk of the MAPK signaling pathway with other proteins and signaling pathways have a crucial impact on clinical outcomes of targeted therapies and plays important role during development of drug resistance in cancers. We discuss cross-talk of the MAPK/ERK signaling pathway with other signaling pathways, in particular interplay with the Hippo/MST pathway. We demonstrate the mechanism of cell death induction shared between MAPK/ERK and Hippo/MST signaling pathways and discuss the potential of combination targeting of these pathways in the development of more effective anti-cancer therapies.

## 1. Introduction

The mitogen-activated protein kinase (MAPK)/extracellular signal-regulated kinase (ERK) signaling pathway represents a highly conserved signal-transduction pathway comprised of three individual kinases, namely RAF, MEK and ERK [1]. MAPK/ERK pathway was primarily described as a transducer of extracellular signals from epidermal growth factor receptor (EGFR) to ERK1/2 kinase [2]. ERK1/2 kinase phosphorylates several protein substrates which participate in the regulation of basal cellular functions involving survival, differentiation and metabolism. Deregulation of the MAPK/ERK signaling occurs in several types of cancers rendering components of the MAPK/ERK pathway as a potential targets for cancer therapy [3].

Development of resistance in cancer cells represents major problem during monotherapy with a single MAPK/ERK inhibitor. Resistance development often arises as a result of the MAPK/ERK cross-talk with other signaling pathways such as the PI3K/AKT/MTOR signaling pathway [4]. On the other hand, several reports describe a crucial role for activation of the MAPK/ERK signaling during cell death induction in broad spectrum of cancer cells suggesting tumor-suppressor activity of the MAPK/ERK signaling. Components of the Hippo/MST signaling pathway were recently demonstrated as targets of MEK and ERK kinase providing evidence for tumor-suppressor activity of the MAPK/ERK signaling in cancer cells.

In this review, we tend to discuss the impact of the MAPK/ERK cross-talk with other signaling pathways and MAPK/ERK protein interactors on the regulation of individual cellular processes in cancer cells. We focus mainly on cross-talk between MAPK/ERK, Hippo/MST and PI3K/AKT/MTOR signaling pathways in various cancer cells and discuss interplay between these pathways in the context of current cancer therapies. We highlight similarity between MAPK/ERK and Hippo/MST signaling pathway in regulation of proliferation and induction of cell death in cancer cells. Finally, we discuss combination targeting of MAPK/ERK-Hippo/MST-PI3K/AKT/MTOR signaling pathways for improve outcomes of current cancer therapies.

## 2. Canonical MAPK/ERK Signaling Pathway

MAPK1/ERK2 and MAPK3/ERK1 represent two well-known serine/threonine kinases involved in the modulation of the MAPK signal transduction pathway. ERK1 and ERK2 kinase acts as the final signal node of the MAPK/ERK signaling pathway and participates in the regulation of key cellular processes such as proliferation, metabolism, differentiation and survival. ERK1/2 kinase phosphorylates broad spectrum of protein substrates which are involved in transcription regulation, cytoskeleton organization, protein phosphorylation, protein translation and cell death induction. The canonical MAPK/ERK signaling pathway is often demonstrated as a positive regulator of cellular proliferation, growth and survival. Selective inhibition of individual kinases of the MAPK/ERK signaling pathway using targeted inhibitors represents a promising method of effective cancer treatment, however, resistance development seems to be serious problem [4,5].

## 3. Identity and Function of Known ERK Kinase Interactors

**Figure 1:**
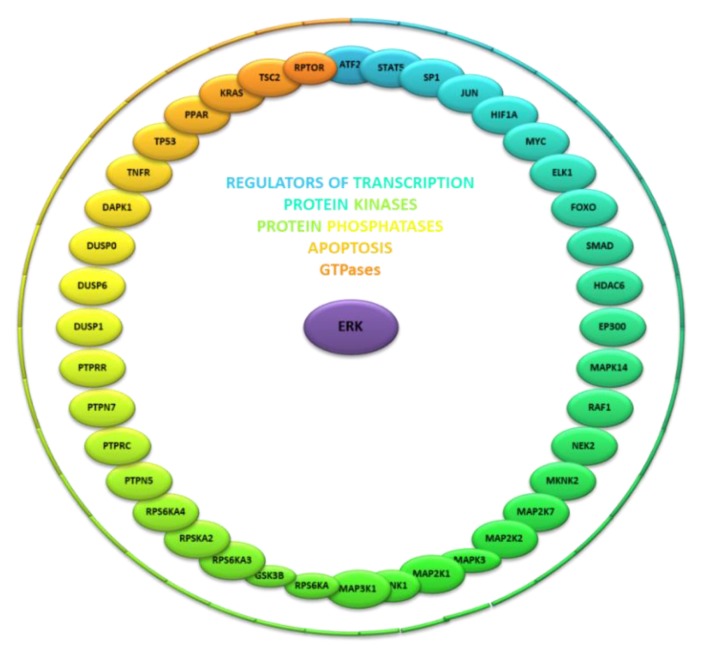
Extracellular signal-regulated kinase (ERK) protein interactors and processes regulated by these interactors.

ERK1/2 kinase shares large number of protein interactors. BioGRID database contains 270 unique interactors of ERK2 kinase [6]. However, the association of several interactors with ERK2 kinase came from results obtained using high-throughput (HT) techniques or hit only in single evidence. Filtration of interactors based on less than three evidences and interactors tagged as HT mined about 50 protein interactors representing established targets of the ERK kinase (Figure 1). These interactors involve regulators of transcription (ATF2, STAT5, SP1, JUN, HIF1A, MYC, ELK1, FOXO3, SMAD1, HDAC and EP300), protein kinases (MAPK14, RAF1, NEK2, MKNK2, MAP2K7, MAP2K2, MAPK3, MAP2K1, MKNK1, MAP3K1, RPS6KA1, GSK3B, RPS6KA3, RPS6KA2 and RPS6KA4), protein phosphatases (PTPN5, PTPRC, PTPN7, PTPRR, DUSP1, DUSP6 and DUSP9), regulators of apoptosis (DAPK1, TNFRS1A, TP53 and PPARG) and GTPase activity-linked proteins (KRAS, TSC2 and RPTOR). Regulation of these protein interactors by ERK kinase provides diverse outputs on cellular functions.

Activation of ATF2 by ERK kinase promotes growth of intrahepatic cholangiocarcinoma and induces apoptosis in MDA-MB-435 breast cancer cells [7,8]. A decrease in phosphorylation levels of STAT5 and ERK during imatinib treatment promotes lymphopenia in chronic myelogenous leukemia (CML) patients. Inhibition of the ERK/ELK1/HIF1A/VEGFA pathway suppresses angiogenesis in colorectal cancer [9]. Several reports demonstrate activation and stabilization of MYC transcription factor by ERK-dependent phosphorylation as oncogenic mechanism responsible for proliferation and metabolic reprogramming in broad spectrum of cancer cells [10,11,12]. Suppression of FOXO3 and ERK phosphorylation during glucosamine treatment decreases proliferation of A549 cancer cell [13]. Ursolic acid inhibits proliferation of colon cancer cells through inhibition of ERK phosphorylation and attenuation of EP300-mediated acetylation of NF-κB and CREB2 [14]. Members of the RPS6K family were recently identified as synthetic lethal targets for combinatory treatment with rapalog and inhibitors of the MAPK/ERK signaling in triple-negative breast cancer cells demonstrating ERK kinase as a regulator of protein translation [15]. Inhibition of the MAPK/ERK signaling abolished the induction of apoptosis triggered by GSK3B inhibitors in acute promyelocytic leukemia treated with lithium [16]. Inhibition of DUSP phosphatase using BC1 inhibitor resulted in ERK kinase hyperactivation and necrosis in malignant peripheral nerve sheath tumors [17]. On the other hand, PTPN phosphatase promotes progression of glioma by activating ERK signaling [18]. A germ line mutation in DAPK1 death domain which disturbs stabile interaction with ERK kinase inactivates apoptosis induced by ERK [19]. Activation of the MAPK/ERK signaling by hispidulin inhibits hepatocellular carcinoma growth and metastasis through the regulation of PPARG [20]. Receptor for hyaluronan-mediated motility (RHAMM) activates ERK kinase in a YAP-dependent manner and modulates breast cancer cell motility [21]. Interaction with KRAS suggests feedback loop regulation of ERK signaling and interaction with TSC2 and RPTOR represents potential cross-talk with the MTOR signaling pathway.

## 4. Identity and Function of Interactors Shared Between ERK and MST Kinases

**Figure 2:**
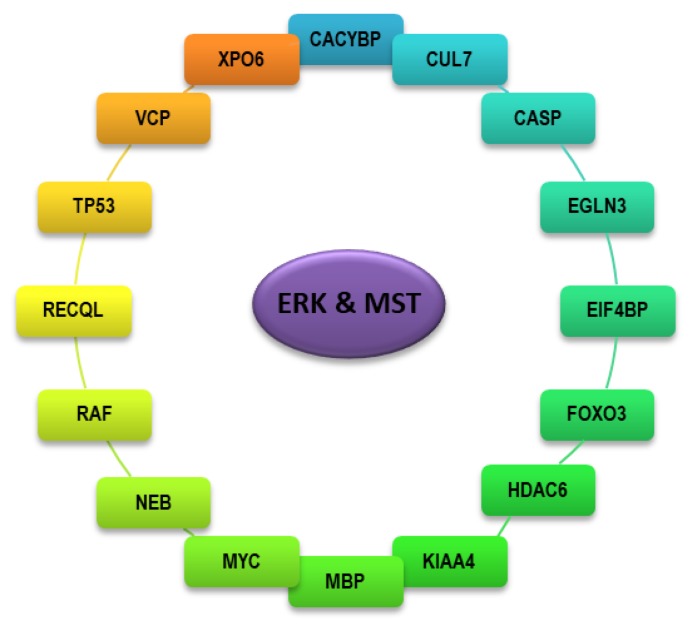
Protein interactors shared between ERK and MST kinase.

ERK kinase shares several protein interactors with the MST kinase representing the central kinase of the Hippo signaling pathway (Figure 2). Activity of these interactors can be regulated in a positive or negative manner. CACYBP (calcyclin-binding protein) represents part of ubiquitin E3 complexes and participates in calcium-dependent ubiquitination and proteasomal degradation of target substrates which plays a role during the regulation of proliferation in several cancer cells [22,23]. Interaction with CACYBP represents a potential mechanism of ERK and MST kinase regulation by ubiquitin-dependent degradation. CUL7 (cullin-7) represents another ERK and MST kinase interactor involved in the process of protein ubiquitination. CUL7 is a core part of 3-M complex required during regulation of cytoskeleton dynamics and genome integrity identified as a cause of hereditary human growth retardation syndrome. Activity of CUL7 is regulated by MTOR signaling providing new evidence for the regulation of ERK and MST kinase through MTOR signaling [24,25]. Anti-apoptotic protein BIRC5 was described as a target of CUL7 involved in regulation of apoptosis [26]. Since MST kinase represents a known regulator of genome integrity during apoptosis, interaction with CUL7 shed new light on this process together with involvement of the ERK kinase which was not previously demonstrated. VCP (Transitional endoplasmic reticulum ATPase) promotes protein sorting at the level of the Golgi apparatus where it acts as a part of higher-order regulatory complexes. VCP participates in the final step of ubiquitination and endoplasmic reticulum-associated degradation of HMGCR (3-hydroxy-3-methylglutaryl-coenzyme A reductase) in a sterol-dependent manner [27]. HMGCR represents a rate-limiting enzyme of the cholesterol biosynthesis pathway which was previously reported as a regulator of the Hippo/MST signaling; however, the role of the ERK kinase in this process remains unclear. EGLN3 (egl nine homolog 3, part of a family of proline hydroxylases) represents an important cellular oxygen sensor participating in the post-translational regulation of the HIF (hypoxia-inducible factor) suggesting the potential link between cellular respiration, ROS (reactive oxygen species) production and regulation of ERK and MST kinase [28]. EGLN3 induces apoptosis in cardiomyocytes and neurons through disruption of the BAX-BCL2 complex followed by caspase-3 activation providing plausible evidence for cooperation between ERK and MST kinase during regulation of apoptosis [29,30]. Hydroxylation of PKM (pyruvate kinase M) by EGLN3 activity limits glycolysis during hypoxia rendering interaction between EGLN3, ERK and MST as a mechanism responsible for the regulation of the metabolism [31,32]. A dephosphorylated form of the EIF4EBP2 (eukaryotic translation initiation factor 4E-binding protein 2) binds to EIF4E and prevents EIF4F complex assembly, and hence acts as a repressor of translation. Phosphorylation of EIF4EBP2 triggers dissociation from EIF4E leading to translation initiation [33]. ERK and MST kinase are well known regulators of translation rendering EIF4EBP2 as a target of both kinases. Moreover, EIF4EBP2 is phosphorylated also by the PI3K/AKT/MTOR signaling pathway providing more evidence for tight cooperation between MAPK/ERK, Hippo/MST and PI3K/AKT/MTOR signaling pathways [34]. MBP (myelin basic protein) is a component of the neuronal myelin membrane which regulates processes such as the re-myelination of axons. MBP also acts as a regulator of T cells proliferation [35]. Interaction of ERK and MST kinase with MBP sheds new light on the role of these kinases in the regulation of neuron cells and immunity function. Nebulin binds and stabilizes F-actin filaments and is responsible for the structural integrity of the cell [36]. Role for MST kinase in mechanotransduction was already characterized however participation of ERK kinase in this process remains obscure [37]. Interaction of ERK and MST kinase with XPO6 (exportin 6) represents mechanism supporting nuclear-cytosol shuttling of ERK and MST kinase and highlights the role of these kinases in the RNA metabolism [38]. FOXO3, MYC and TP53 proteins represent common targets of ERK and MST kinase participating in cellular processes such as regulation of cellular metabolism, proliferation and apoptosis. The role of these proteins in ERK and MST signaling will be discussed later. HDAC6 is a member of the family of histone deacetylases participating in the deacetylation of lysine residues on the N-terminal part of the core histones [39]. Moreover, participation of HDAC6 in the regulation of the protein acetylation of cytosolic proteins was also described [40]. Regulation of the MST signaling by acetylation/deacetylation was previously described [41]. The role of ERK kinase in this process remains unknown. The interaction of ERK and MST kinase with the activity of caspases (caspase -3, -7, -8, -9) represents one of the crucial mechanisms of cell death regulated by these kinases, as will be discussed later.

## 5. Cross-Talk of the MAPK/ERK Pathway with Other Signaling Pathways

ERK kinase regulates the upstream components of the MAP kinase signal transduction pathway as well as its own activity by oneself in feedback loops occurring in internal or external, positive or negative, regulatory feedback loops. In internal negative feedback loops, ERK abrogates SOS (Son of sevenless) complex formation and in internal positive feedback loops it stimulates downstream interactors of growth factor signaling at the growth factor receptor level. In external negative feedback loops ERK inhibits EGFR (epidermal growth factor receptor) signaling and in external positive feedback loops it stimulates the activity of growth factor signaling through external factors. This model can partially explain bistability and oscillations in the ERK signaling as demonstrated using theoretical dynamic modelling performed by Arkun et al. [42]. MTORC1 signaling represents another pathway regulated by ERK kinase. ERK kinase directly phosphorylates TSC2 at S664 leading to TSC2 activation. TSC2 acts as a GTPase-activating protein for the small GTPase RHEB, a direct activator of the MTORC1 kinase which in turn activates S6K and inhibits 4EBP1 resulting in stimulation of protein translation and anabolic metabolism [43,44,45]. Contrary to this, inhibition of TSC2 activity through phosphorylation by RPS6KA/RSK at S1798 was also documented [46]. RPS6KA/RSK represents an established kinase directly phosphorylated and activated by ERK kinase but shows the opposite effect on TSC2 regulation. A decrease in S6K phosphorylation at the activatory S424 after ERK and RPS6KA/RSK kinase activation during treatment with PI3K inhibitor GDC-0941 was recently documented [47]. These results stress potential cross-talk of ERK and PI3K/AKT with other signaling pathway(s) in the process of MTORC1 and S6K kinase activation. GSK3B represents another kinase regulated directly by ERK kinase. ERK kinase directly binds and phosphorylates GSK3B at T43 which resulting in inactivation of GSK3B and increase in glucose metabolism which is in agreement with effect observed after phosphorylation of GSK3B by RPS6KA/RSK [48,49]. These results provide another evidence for biphasic behavior and oscillations in ERK signaling. A detailed overview of proteins and amino acid residues phosphorylated by the MAPK/ERK signaling pathway can be found in PhosphoSitePlus database [50].

## 6. Mechanisms of Cell Death Induced by the MEK/ERK Kinase 

Inhibition of the MEK/ERK signaling blocks proliferation and induces apoptosis in cancer cells. Contrary to this, several reports demonstrate activation of MEK/ERK signaling as a key apoptotic mechanism triggered by anti-cancer drugs in cancer cells [51,52]. These observations usually build on the fact that a specific blockade of the MEK/ERK signaling interferes with tumor growth inhibition and cell death induction triggered by various drugs in cancer cell models. Induction of apoptosis by cisplatin in HeLa and HL-60 cells was dependent on activation of the ERK signaling and NOXA expression. Inhibition of ERK signaling through MEK inhibitor U0126 and ERK inhibitor peptide II abrogated NOXA expression and apoptosis induced by cisplatin. Moreover, a decrease in levels of anti-apoptotic proteins MCL1 and BCL-XL was described as ERK-dependent [53]. The transient expression of H-RAS^V12^ in HEK293T cells induces NOXA expression and BECLIN1 autophagic cell death in an ERK-dependent manner [54]. Recovery of ERK signaling was essential for heavy ion irradiation-induced multiple caspase activation during glioma cell death. Activation of ERK signaling together with the activation of caspase-3, -8, -9 induces apoptosis in T98G and U251 glioma cells after heavy ion irradiation and the activation of caspases was diminished by expression of the dominant-negative from of ERK kinase in these cell lines [55]. Activation of caspase-3, -7, -8, -9 in an ERK-dependent manner was recently documented in T-ALL cells treated with PI3K inhibitor GDC-0941 [47]. Galectin-3 activation of caspase-9 and induction of apoptosis in T-cells was also dependent on activation of the ERK signaling [56]. ERK and NOTCH signaling were described as regulators of caspase-3 activation and apoptosis induction in endothelial cells during chronic obstructive pulmonary disease [57]. Activation of ERK kinase and inhibition of AKT kinase activates caspase-3 in several models of melanoma treated with ACA-28 modulator [58]. Activation of ERK expression by venom toxin bengalin induced caspase-3 activation and apoptosis in U937 leukemic cells [59]. A decrease in the expression of BCL-XL and MYC protein, activation of caspase-3, -8, -9 and apoptosis triggered by natural alkaloid tryptanthrin, depends on the activation of ERK signaling in several human leukemia cells [60]. Cross-talk between caspase-8, reactive oxygen species and ERK signaling was elucidated as a mechanism modulating the fate of phagocytosing neutrophils [61]. Activation of an inducible form of RAF in HEK293 cell resulted in the ERK signaling stimulation and caspase-8 activation [62]. A critical role for caspase-8 in EGF signaling was also described [63]. Cytotoxicity of thujaplicin in hepatocellular carcinoma was also dedicated to the activation of the ERK signaling [64]. Inhibition of the ERK signaling abolished alterations in the BCL2 family of proteins expression, the activation of caspase-3, -7 and apoptosis in lung cancer cells treated with quercetin [65]. Hyperactivation of the ERK signaling by multiple mechanisms exerts toxicity to RTK-RAS mutation-driven lung adenocarcinoma cells [66]. Expression of auto-activating ERK2 kinase mutants in prostate cancer cells induces cell cycle arrest independently on RAF signaling [67]. Induction of apoptosis and autophagy in osteosarcoma cells by biphenolic compound honokiol depends on activation of the ERK signaling [68]. ERK signaling plays a critical role during the activation of caspase-3, -8 and autophagic cell death induced by 2-amino-nicotinonitrile (w09) in gastric cancer cells [69]. Sustained ERK activation was essential for the inhibition of proliferation and G0/G1-phase cell-cycle arrest in human carcinoma cells treated with farrerol [70]. ERK also mediates cell-cycle arrest and senescence in prostate neoplasm and blocks transformation of primary cells by oncogenic RAS [71]. Activation of the ERK signaling suppresses growth of antigen-stimulated effector T cells [72]. Biphasic activation of the ERK-MSK1 signaling regulates apoptosis induced by DNA damage [73]. These results render ERK kinase as a potential tumor-suppressor. 

## 7. Mechanisms of Cell Death Orchestrated by the Hippo/MST Signaling Pathway

The Hippo signaling pathway controls development and organ size in diverse species. Deregulation of this pathway induces tumor formations in model organisms and occurs in a broad spectrum of human carcinomas [74]. MST1/2 serine/threonine kinases represent central regulators of the Hippo signaling pathway. Activation of MST1/2 kinase inhibits cellular proliferation and induces apoptosis in several cancer cells as well as in model organisms and hence acts as a tumor-suppressor [75]. Hippo/MST and MAPK/ERK signaling pathways share several mechanisms to regulate cellular proliferation and apoptosis. MST signaling decreases TEAD activity and expression of *MYC* oncogene in T-ALL cells and breast cancer cells as well as in xenograft mice model of breast cancer [76,77]. MST kinase regulates glucose metabolism in T-ALL, laryngeal squamous and breast cancer cells through inhibition of the TEAD transcription activity and expression of GLUT (glucose transporter) [76,78,79]. MST kinase induces apoptosis through expression of pro-apoptotic protein NOXA in several cancer cells through phosphorylation and activation of FOXO (forkhead box O) transcription factors [80,81,82]. MST kinase directly phosphorylates and activates pro-apoptotic protein BIM, caspase-3, -9 and apoptosis in pancreatic beta-cells [83]. Hippo/MST signaling also inhibits expression and activity of several anti-apoptotic proteins such as IAP, MCL1 and BCL-XL [84,85,86]. MST also activates caspases and caspases potentiate MST kinase activity in positive feedback loops. MST signaling was described as a potent activator of caspase-3, -7,-9 and an intrinsic apoptotic pathway through mechanisms discussed above and cleavage of the MST kinase by caspase-3, -7 potentiates its pro-apoptotic activity [87,88,89]. Moreover, activation of caspase-8 by the Hippo/MST signaling was also documented [47,90]. Interplay between caspase-8 and ERK signaling represents one of the key mechanisms in the EGFR signaling pathway as demonstrated by several reports rendering MST as a regulator of this process [55,62,63]. Finally, a computational model predicting diverse dynamic profiles of the Hippo-ERK interaction network was constructed [91].

## 8. Activation of the Hippo/MST Signaling Pathway in Cancer Cells

**Figure 3:**
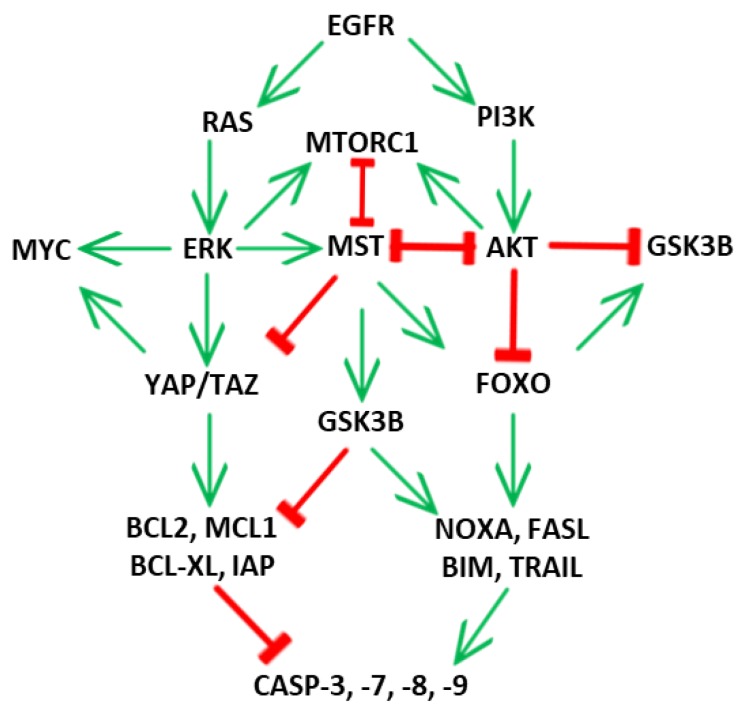
Cross-talk of the mitogen-activated protein kinase (MAPK)/ERK signaling pathway and mechanism of cell death induction through the ERK-Hippo interplay.

Hippo/MST signaling and ERK signaling pathways share several targets to regulate proliferation and cell death in cancer cells (Figure 3). Activation of the Hippo/MST signaling was demonstrated as a crucial mechanism responsible for activity of several anti-cancer compounds. All these results suggest the synergistic effect between inhibitors/activators of ERK signaling and activators of the Hippo/MST signaling for cancer therapy. AKT kinase phosphorylates MST1 at T120 and inhibits MST1 activity [92]. Targeted inhibition of the PI3K/AKT/mTOR signaling axis triggers activation of the MST kinase and inhibits activity of YAP effector in a broad spectrum of cancer cells. Treatment of T-ALL cells with PI3K inhibitor GDC0941 activates MST1 kinase, ERK kinase and apoptosis [47]. LY294002 inhibitor induces suppression of cell growth and apoptosis in castration-resistant C4-2 prostate cancer cells and HCT116 colon cancer cells [92,93]. Wortmannin blocks YAP activation and MYC expression mediated by EGF in hepatocellular carcinoma and mammary epithelial cells [94,95]. The combination of PI3K/mTOR inhibitors with FGFR4 inhibitor BLU9931 potentiates MST1 activation and induces apoptosis in HER2^+^ breast cancer cells [96]. Pan-MTOR inhibitor MLN0128 activates caspase-3, -7 and promotes apoptosis in intrahepatic cholangiocarcinoma induced in mice by YAP over-expression [97]. Rapamycin-derived compound temsirolimus triggers YAP protein degradation by autophagy in human angiomyolipoma [98]. Several natural compounds with anti-cancer activity were described as potent activators of MST kinase in cancer cells. Naphthoquinonic compound shikonin disturbs YAP1-TEAD1 interaction through the activation of MST1 and ERK signaling in T-ALL cells [76,99]. Flavonol fisetin activates LATS and ERK kinase and induces apoptosis in osteosarcoma cells [100]. The polyphenolic compound curcumin induces cell cycle arrest, autophagy and apoptosis through the production of reactive oxygen species (ROS), activation of ERK kinase, MST kinase, caspase-3, -9 and down-regulation of YAP protein in various cancer cell models [101,102,103]. The inhibition of oncogenic Hippo-YAP signaling through the activation of LKB1 tumor suppressor by honokiol abrogates breast tumorigenesis and metastasis in mice [104,105]. Several other drugs and compounds were described as activators of the Hippo/MST signaling in cancer cells. Vitamin E analogues activate MST1 and ERK signaling in T-ALL cells and breast cancer cells leading to apoptosis induction [8,80]. An inhibitor of HMGCR, the rate limiting enzyme of the mevalonate biosynthesis, suppresses malignant mesothelioma cells through blocking of the YAP/CD44 axis [106]. Pyranocoumarin decursin stimulates LATS kinase phosphorylation and YAP protein degradation through activation of TRCP ubiquitin E3 ligase in hepatocellular carcinoma [107]. Tetracyclic triterpene cucurbitacin B induces apoptosis through activation of LATS kinase and caspase-3 in colorectal carcinoma cells [108]. Flavone apigenin disrupts YAP-TEAD interaction and decreases viability and migration of triple-negative breast cancer cells as well as tumor formation in vivo [109].

## 9. Combination Targeting of MAPK/ERK, PI3K/AKT/MTOR and Hippo/MST Pathways in Cancers

**Figure 4:**
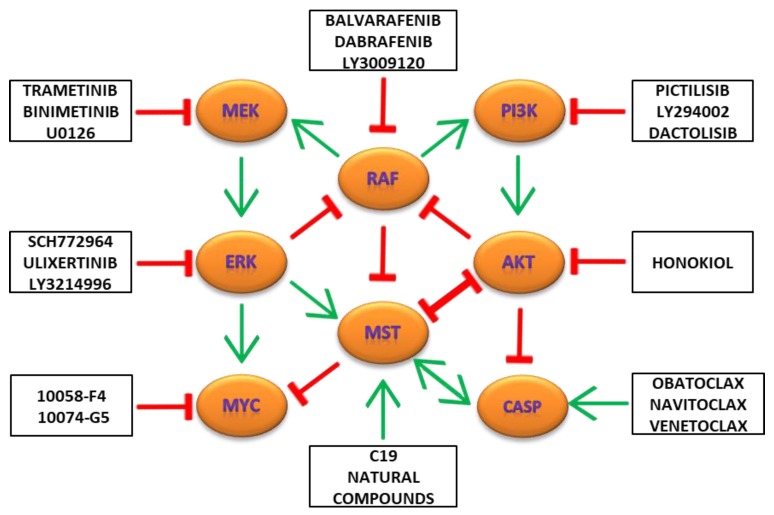
Targeting of the MAPK/ERK-Hippo/MST-PI3K/AKT network for effective cancer therapy.

Targeted inhibition of the MAPK/ERK signaling pathway triggers tumor growth suppression and cell death in cancer cell models. The final effect of the MAPK/ERK regulation depends on actual cross-talk with other signaling pathways active in cancer cells. RAS/RAF signaling activates PI3K/AKT as well as MEK/ERK signaling pathways and could be inhibited by ERK and AKT kinase in feedback loop [110,111,112]. Several targeted inhibitors of RAF kinase have recently undergone clinical testing against various types of cancers (balvarafenib, dabrafenib and LY3009120) [113,114]. However, activation of MEK/ERK kinase by mechanisms alternative to the RAF signaling and development of cancer resistance during monotherapy with single RAF kinase inhibitor often occurs [115]. Hence, combination of RAF-targeted inhibitors with targeted inhibitors of the MEK kinase (trametinib, binimetinib and U0126) and ERK kinase (ulixertinib, SCH772964 and LY3214996) can significantly improve the outcome of cancer therapies (Figure 4) [116,117]. Activation of the Hippo/MST signaling pathway represents an important mechanism of cell death induction during the targeted blockade of PI3K/AKT and MAPK/ERK signaling pathways in various cancer cells [118,119,120]. Several synthetic (C19) and natural compounds were reported as potent activators of the Hippo/MST signaling in broad spectrum of cancer cells. Combination of the Hippo/MST signaling activators with PI3K/AKT and MEK/ERK kinase inhibitors can overcome the development of resistance in cancer cells and boosts therapy efficiency as documented recently [118]. Escape of high-risk neuroblastomas from the selective pressure of MEK inhibition may be sensitive to combination therapies targeting Hippo/MST and MEK signaling [121]. Combination targeting of Hippo/MST and MAPK/ERK signaling pathways represents new therapeutic strategy for uveal melanoma [122]. Activation of the Hippo/MST signaling can be beneficial during inhibition of pancreatic ductal adenocarcinoma development treated with U0126 and LY294002 inhibitors [123]. The activation of the Hippo/MST signaling inhibits YAP-dependent transcription resulting in decreased expression of MYC, BCL2, BCL-XL and MCL1 anti-apoptotic proteins and induction of caspase-dependent cell death. Combination treatment with BH3 mimetics, the specific inhibitors of BCL2, BCL-XL and MCL1 anti-apoptotic proteins (obatoclax, navitoclax and venetoclax), together with specific inhibitors of MYC transcription activity (10058-F4 and 10074-G5) represents another promising strategy to improve targeted therapies based on PI3K/AKT/MTOR and MAPK/ERK inhibitors (Figure 4) [124,125]. Inhibition of MAPK/ERK signaling using PD98059 overcomes resistance to BH3 mimetics obatoclax in small-cell lung cancers [126]. BH3 mimetics reduced viability of melanoma cells relapsed after BRAF/MEK inhibitors treatment [127]. BH3 mimetics synergize with the MEK kinase inhibitor U0126 during apoptosis induction in melanoma cells [128]. BH3 mimetics gossypol activates ERK signaling and apoptosis in malignant mesothelioma (MM) cells as well as in mice intraperitoneally transplanted with mouse MM cell lines [129]. Activation of the MEK/ERK signaling upon PI3K signaling inhibition was reported to induce cell death in various cancer cells [130]. MEK/ERK signaling pathways acts as a potential tumor suppressor regulating proliferation and cell death in cancer cells [131]. Hyperactivation of ERK signaling inhibits proliferation and induces apoptosis in a variety of BRAF (V600E) cancer cells [132]. In such cases, targeted inhibition of the MEK/ERK signaling suppresses cell death and potentiates the development of resistance in cancer cells [133]. Hence, a rational combination of the MAPK/ERK and PI3K/AKT/MTOR inhibitors with activators of the Hippo/MST signaling, BH3 mimetics and inhibitors of MYC transcription activity can boost current targeted therapies. 

## 10. Conclusions

Dual-face activity of the MAPK/ERK signaling has a significant impact on clinical outcomes of the MAPK/ERK targeted therapies. The combination of PI3K/AKT/MTOR and MEK/ERK inhibitors can potentially decrease therapy efficiency due to different impacts on the Hippo/MST signaling pathway. While active PI3K/AKT/MTOR signaling has a negative impact on the Hippo/MST activity, activation of the MEK/ERK signaling potentiates the induction of cancer cell death through the Hippo/MST signaling pathway. Based on this evidence, a combined inhibition of the MAPK/ERK pathway at the levels of RAF and MEK/ERK kinase seems to be effective in cancer cells depending exclusively on the activity of the MAPK/ERK signaling pathway. Reactivation of the MEK/ERK axis in cancer cells addicted to inhibition of the Hippo/MST pathway through the PI3K/AKT/MTOR signaling pathway can be beneficial for therapy efficiency after targeted PI3K blockade. Moreover, activation of the Hippo/MST signaling and chemical regulation of its downstream targets can significantly boost clinical outcomes of targeted therapies based on MAPK as well as PI3K signaling inhibitors. Detailed characterization of the MAPK/ERK regulatory network brings also perspectives for the treatment of several other pathologies such as metabolic syndromes, rare genetic diseases and neurodegenerative diseases [134].
